# Exosomes Isolated from Ascites of T-Cell Lymphoma-Bearing Mice Expressing Surface CD24 and HSP-90 Induce a Tumor-Specific Immune Response

**DOI:** 10.3389/fimmu.2017.00286

**Published:** 2017-03-16

**Authors:** Florencia Menay, Leticia Herschlik, Julieta De Toro, Federico Cocozza, Rodrigo Tsacalian, María José Gravisaco, María Paula Di Sciullo, Alejandrina Vendrell, Claudia I. Waldner, Claudia Mongini

**Affiliations:** ^1^Centro de Estudios Farmacológicos y Botánicos-Consejo Nacional de Investigaciones Científicas y Técnicas (CEFYBO-CONICET), Facultad de Medicina, Universidad de Buenos Aires, Buenos Aires, Argentina; ^2^Instituto Nacional de Tecnología Agropecuaria (INTA), Buenos Aires, Argentina

**Keywords:** exosomes, T-cell lymphoma, immune response, tumor vaccine, ascites

## Abstract

Extracellular vesicles (EVs), including endosome-derived nanovesicles (exosomes), are involved in cell–cell communication. Through transfer of their molecular contents, extracellular nanovesicles can alter the function of recipient cells. Due to these characteristics, EVs have shown potential as a new alternative for cancer immunotherapy. Tumor exosomes isolated from malignant ascites can activate dendritic cells, thereby priming the immune system to recognize and kill cancer cells. However, a suppressive role on tumor immune response has also been reported, suggesting that the neoplastic stage of carcinogenesis and the microenvironment where tumor cells grow may influence the amount of EVs released by the cell. This neoplastic stage and microenvironment may also impact EVs’ components such as proteins and miRNA, determining their biological behavior. Most T-cell lymphomas have an aggressive clinical course and poor prognosis. Consequently, complementary alternative therapies are needed to improve the survival rates achieved with conventional treatments. In this work, we have characterized EVs isolated from ascites of mice bearing a very aggressive murine T-cell lymphoma and have studied their immunogenic properties. Small EVs were isolated by differential centrifugation, ultrafiltration, and ultracentrifugation at 100,000 × *g* on a sucrose cushion. The EVs were defined as exosomes by their morphology and size analyzed by electron microscopy, their floating density on a sucrose gradient, as well as their expression of endosome marker proteins ALIX, TSG-101; the tetraspanins CD63, CD9, and CD81. In addition, they contain tumor antigens, the marker for malignancy CD24, the heat shock protein HSP-70, and an unusual surface expression of HSP-90 was demonstrated. The administration of EVs isolated from ascites (EVs A) into naïve-syngeneic mice induced both humoral and cellular immune responses that allowed the rejection of subsequent tumor challenges. However, the immunization had no effect on a non-related mammary adenocarcinoma, demonstrating that the immune response elicited was specific and also it induced immune memory. *In vitro* analysis demonstrated that T-cells from EVs A-immunized mice secrete IFN-γ in response to tumor stimulation. Furthermore, tumor-specific CD4+ and CD8+ IFN-γ secreting cells could be efficiently expanded from mice immunized with EVs A, showing that a T helper 1 response is involved in tumor rejection. Our findings confirm exosomes as promising defined acellular tumor antigens for the development of an antitumor vaccine.

## Introduction

Extracellular vesicles (EVs) are cell-derived vesicles of different sizes and intracellular origins, including small EVs formed inside endosomal compartments and EVs of various sizes budding from the plasma membrane (1–3). Exosomes are 50–100 nm size EVs, originated from late endosomes, which are actively exocytosed to the extracellular medium (2). These bioactive nanovesicles, whose function is to promote intercellular communication, have been isolated from very different cell types including tumor cells. Although the sources of exosomes are different, they have common characteristics such as the presence of a lipid-bilayer structure containing microRNAs, mRNAs, DNA fragments, and proteins. They are also similar in size and density. Exosomes express certain marker molecules: proteins that designate an endosome origin (ALIX, TSG-101) and tetraspanins (CD9, CD63, CD81) (3). In addition, others molecules generally found in exosomes are heat shock proteins (HSP-60, HSP-70, and HSP-90); proteins involved in antigen presentation, e.g., MHC I and MHC II; and particularly, exosomes derived from tumors express tumor antigens (2, 4).

Direct interaction between tumor cells and their environment is essential for cancer progression. To achieve this communication, effective cross talk is required. Over the last years, it has been demonstrated that EVs and exosomes play a critical role in cell–cell communication. For example, exosomes are very important to induce a pro-carcinogenic microenvironment and regulate the immune response aimed at promoting tumor progression and survival. To accomplish such survival, exosomes remodel the extracellular matrix, inducing angiogenesis, thrombosis, and proliferation of tumor cells (5–8). In addition, tumor-derived exosomes interact extensively with immune cells (9). Exosomes have been demonstrated to activate dendritic cells (DCs), thereby priming the immune system to recognize and kill cancer cells (4, 9, 10). However, a suppressive role on tumor immune response has also been reported. For example, exosomes may express Fas-L (11) or galectin (12), which are responsible for the induction of apoptosis in activated T-lymphocytes, the inhibition of DCs maturation (13), the impairment of the lymphocyte response to IL-2 (14), the downregulation of the cytolytic activity of NK-cells (15), the generation of regulatory T-cells (16), and the blockage of the binding of tumor-specific antibodies to tumor cells to reduce antibody-dependent cell cytotoxicity reactions (17). Furthermore, the neoplastic stage of carcinogenesis and the microenvironment where tumor cells grow may influence not only the amount of EVs released but also the expression of the molecules in the vesicles and consequently, their biological behavior.

The potential of EVs and specially exosomes in the immunotherapy of cancer is already being evaluated in clinical trials (18, 19). Nevertheless, the contradictory results obtained so far in relation to the effectiveness of exosome stimulation on the immune system constrain their use. Recent studies have shown that exosomes can suppress or induce antigen-specific tolerance as well as prime an immune response when injected into naïve recipients (20). Despite the vast advances in knowledge of EVs and exosomal biology, various issues are not yet fully understood. Therefore, to employ exosomes for cancer vaccines, the expression of different proteins as well as the immune response induced by them need to be characterized thoroughly, to ensure that they will achieve the desired immunostimulation.

Exosomes derived from B-cell lymphomas already have been characterized extensively (21–23); however, there are only a few reports describing T-cell lymphoma-derived exosomes. Most T-cell lymphomas have an aggressive clinical course and a poor prognosis. Consequently, alternative or complementary therapies are needed to improve the survival rate already achieved with conventional treatments.

In this study, we have characterized the EVs derived from the ascites of a very aggressive murine T-cell lymphoma (EVs A) by their size, morphology, floating density on a sucrose gradient, and the expression of marker-proteins ALIX, TSG-101, CD63, CD81, and CD9. In addition, we have investigated their ability to modulate tumor-specific immune response by the administration of exosomes into syngeneic mice. We have demonstrated that EVs A induced both humoral and cellular immune responses. The immune response elicited is particularly important in that it is specific and induces immune memory; for it failed to reject a non-related mammary adenocarcinoma but allowed the rejection of subsequent tumor challenges with the same tumor that have originated the EVs. Overall, our results demonstrate the ability of endosome-derived EVs secreted by an advanced-stage T-cell lymphoma, to stimulate an immune specific response.

## Materials and Methods

### Cell Culture

The syngeneic BALB/c T-cell lymphoma cell line, LBC (H-2^d^) was maintained in RPMI 1640 (Gibco™, Invitrogen, CA, USA) supplemented with 10% heat-inactivated, fetal-bovine serum (FBS) (Internegocios, Mercedes, Buenos Aires, Argentina), 2mM glutamine, 25mM HEPES buffer, 100 IU/ml penicillin, 100 μg/ml streptomycin, and 0.05 mM 2-mercaptoethanol, as previously described (24).

### Mice

Six- to ten-week-old female immunocompetent BALB/c mice were purchased from the School of Veterinary Sciences, Universidad de Buenos Aires (Buenos Aires, Argentina). Animals were fed on Cargill pellets and water *ad libitum*. All animal procedures were conducted in accordance with Institutional Animal Care and Use Committee (CICUAL) guidelines of University of Buenos Aires Medical School.

### EVs Isolation

For the isolation of EVs from ascites (EVs A), BALB/c mice were inoculated with 1.0 × 10^6^ LBC cells i.p. At 19 ± 2 days, the mice were anesthetized with a combination of ketamine (100 mg/kg) and xylazina (10 mg/kg) by i.p. injection. The ascitic fluid was obtained by ventral puncture. EVs from LBC-cell culture (EVs C) were obtained from bovine-EVs-depleted medium after 48 h of LBC-cell culture at 37°C and 5% CO_2_ atmosphere. EVs were isolated as previously described (25). Briefly, ascites or LBC-conditioned medium were centrifuged at 300 × *g* for 10 min to separate floating cells. Supernatants were removed and centrifuged successively at 800 × *g* for 30 min, 10,000 × *g* for 30 min. Supernatants were filtered through a 0.22 μm porous membrane and centrifuged at 100,000 × *g* for 1 h on a 30% sucrose weight/volume (w/v) cushion. EVs contained in the 30% sucrose cushion were resuspended in phosphate-buffered saline (PBS) and were concentrated in a last step of 1 h ultracentrifugation at 100,000 × *g*.

The EVs in the pellet were resuspended in PBS and kept at −80°C. The amount of exosomal proteins recovered was determined by the Bradford method (25).

### Cell Lysates

#### Protein LBC-Cell Lysates (LBC p-Lysate)

Protein LBC-cell lysates (LBC p-lysate) were obtained by incubating cell suspensions with a lysis buffer for 20 min on ice and centrifuged as previously described (25).

#### Freeze–Thaw Lysates (LBC Lysate)

LBC cells were harvested and washed twice with PBS. Cells were then suspended in PBS (2.0 × 10^6^ cells/ml) and lysed by means of three to four cycles of freezing at −70°C and thawing at 37°C, vortexing after each round. Total cell death was confirmed by trypan blue dye exclusion. Protein concentration was determined by the Bradford method. Lysates were then aliquoted and stored at −70°C until used.

### Cell Immunofluorescence Staining and Flow Cytometry

LBC-cell staining was performed as previously described (24). The following antibodies were employed: anti-mouse CD-24-PE, anti-CD8-PE MAbs, and isotype-matched controls (eBioscience, CA, USA). After incubation with anti-MHC I-Biotin primary antibody, the cells were washed and then incubated with streptavidin–phycoerythrin (PE) conjugated (eBioscience, CA, USA). Fluorescence was measured in a BD FACSCalibur flow cytometer (BD Biosciences, San Jose, CA, USA). The data were analyzed with the Flowing 2.5.1 software.

### EVs Immunofluorescence Staining and Flow Cytometry

Five micrograms of EVs A were incubated with 4-μm diameter aldehyde/sulfate latex beads (Invitrogen, CA, USA), as detailed in Ref. (25, 26). Bound EVs were spun down, and the unoccupied sites were saturated with 100 mM glycine. Then, the EVs were incubated with anti-mouse CD24-PE, anti-CD8-PE, and isotype-matched controls (eBioscience, CA, USA). After incubation with anti-MHC I-Biotin, CD9-Biotin, CD81-Biotin (eBioscience, CA, USA), anti-HSP-70, and anti-HSP-90 (Santa Cruz Biotechnology, TX, USA) primary antibodies, the EVs-coated beads were incubated with a streptavidin–PE-conjugated (Invitrogen, CA, USA) or an Alexa647-conjugated secondary antibody, respectively (Jackson Immuno Research, PA, USA). Fluorescence was measured in a BD FACSCalibur flow cytometer (BD Biosciences, CA, USA). The data were analyzed with the Flowing 2.5.1 software.

### Electron Microscopy

To visualize the EVs’ preparations by electron microscopy, EVs were fixed with 4% paraformaldehyde and loaded into electron microscopy grids (25). Samples were then observed in a transmission electron microscope Phillips Tecnai-10. Vesicles size was assessed with analysis ImageJ analysis software.

### Sucrose Gradient

The floatation of EVs released by LBC cells was performed in a continuous sucrose gradient as described for exosomes (25). Gradient fractions (0.2 ml each) were diluted in 4 ml PBS, centrifuged for 1 h at 100,000 × *g*, resuspended in Laemmli sample buffer, separated on a 10% SDS-PAGE, and transferred onto a nitrocellulose membrane (Hybond-ECL nitrocellulose membrane, 0.2-μm transfer membrane; GE Amersham Life Sciences, USA). Specific proteins then were detected as explained for western blot.

### SDS-PAGE and Western Blot

Ten micrograms of EVs or cell lysates were resuspended in Laemmli sample buffer and separated by electrophoresis on 10% SDS-polyacrylamide gels. Gels were electroblotted onto nitrocellulose paper (Hybond-ECL nitrocellulose membrane, 0.2-μm transfer membrane; GE Amersham Life Sciences, USA) and blocked overnight with 5% non-fat dry milk in Tris buffer saline (B-TBS). Blots were incubated overnight at 4°C with mouse anti-CD63, anti-TSG-101, anti-ALIX, anti-HSP-70, or anti-HSP-90 antibodies (Santa Cruz Biotechnology, TX, USA). Finally, blots were washed and incubated with an anti-mouse HRP-conjugated or an anti-rabbit HRP-conjugated serum. Membranes were revealed with the ECL kit (GE, Amersham Life Sciences, USA) following the manufacturer’s instructions. The GE Healthcare, Image Quant TM-RT ECL, Version 1.0 software was used to detect specific proteins (25).

### Dot Blot

One mircoliter of LBC EVs, or LBC-cell lysate samples, or 2 μl of LBC-cell samples containing 1 μg of EVs or cell lysates and 10,000 LBC cells, respectively, were spotted onto a nitrocellulose membrane (Hybond-ECL nitrocellulose membrane, 0.2-μm transfer membrane; GE Amersham Life Sciences, USA). Membranes were blocked and incubated overnight at 4°C using different dilutions (from 1:200 to 1:6,400) of sera from naïve mice or from mice immunized LBC-cell lysate. Blots were washed and incubated with anti-mouse HRP-conjugated sera for 1 h. Specific proteins were detected as explained for western blot.

### Mice Immunization and Tumor Challenge

Mice were randomly divided into groups and vaccinated i.p. once a week for 2 weeks. For each injection, each group of mice received 10 μg EVs A/ml/mouse, EVs C, or PBS alone as control. Seven days after the last immunization, animals were challenged with 1.0 × 10^6^ LBC tumor cells i.p., which is a dose that has been determined to cause 100% lethality 21 ± 4 days after injection. Mice survival was monitored daily, and the mortality rate and survival were recorded (27).

To evaluate whether mice developed a memory response, mice vaccinated with EVs A, EVs C, or LBC lysate that had rejected the first tumor challenge were rechallenged with 1.0 × 10^6^ LBC tumor cells i.p. 30 days after the first tumor injection. The survival time of animals was recorded daily. Survival curves from these treated mice were compared with survival curves from the naïve-age-matched mice that had been inoculated i.p. with 1.0 × 10^6^ LBC cells at the same time.

To assess the specificity of the immune response generated, EVs A or EVs C-immunized mice that had rejected the LBC tumor and naïve mice were inoculated with 2.0 × 10^5^ LM3 mammary adenocarcinoma cells subcutaneously into the right flank (28). The mice developed a palpable, solid tumor in the inoculated region tumor on day 11 postinoculation. The animals were sacrificed when the tumor reached a volume of 1 cm^3^, approximately 40 days postinoculation.

### Carboxyfluorescein Succinimidyl Ester (CFSE) Proliferation Assay

BALB/c mice were immunized with LBC-cell lysate once a week for 2 weeks. On day 14, spleen cells were obtained under sterile conditions. The proliferative behavior of the lymphocytes was quantified by the CFSE dilution. The CFSE staining was performed before seeding, using the CFSE (Invitrogen, CA, USA) at a final concentration of 5 μM for 10 min at room temperature, followed by immediate quenching with culture medium. CFSE stained splenocytes (1.0 × 10^6^/ml) were seeded in a 24-well plate and incubated during 5 days with 10 μg of EVs, alone; 10 μg of LBC lysate, alone; and 10 μg of EVs A and 1 μg of concanavalin A (CON A), together in the same well. For the positive and negative controls splenocytes were incubated with 1 μg of CON A and RPMI complete medium, respectively. To analyze T-cell subpopulations, stimulated and CFSE stained splenocytes were incubated with PE-conjugated anti-CD8 or anti-CD4 monoclonal antibodies. *In vitro* stimulated splenocytes were analyzed by flow cytometry. Fluorescence was measured in a BD FACSCalibur flow cytometer (BD Biosciences, CA, USA). The data analysis was performed with the Flowing 2.5.1 software (29). Relative proliferative index (RPI) is defined as the ratio between the percentages of stimulated cells by the percentage of control cells.

### Intracellular Staining for IFN-γ

Spleens cells were obtained from naïve mice or immunized with EVs A once a week for 2 weeks. Splenocytes (1.0 × 10^6^/well) were seeded in a 96-well plate. After stimulation with 10 μg of EVs A or 10 μg of LBC lysate during 48 h, splenocytes were incubated with Golgi Stop^®^ (Monensin, BD Biosciences, CA, USA), according with manufacturer’s recommendations, for the final 6 h, and centrifuged at 250 × *g* for 5 min. Cells were resuspended in staining buffer (PBS supplemented with 0.1% sodium azide and 5% FBS, pH 7.4–7.6), and the intracellular staining for IFN-γ expression was accomplished as previously detailed (30). Double-color surface staining was first performed with fluorescein isothiocyanate-conjugated anti-CD4 and PE-conjugated anti-CD8 monoclonal antibodies, and then cells were fixed with 4% paraformaldehyde and permeabilized with permeabilization buffer (PBS supplemented with 0.1% sodium azide, 1% FBS, and 0.1% saponin) and stained with an allophycocyanin-labeled anti-IFN-γ mAb (eBioscience, USA). Samples were acquired in a FACSCalibur flow cytometer (BD Biosciences, San Jose, CA, USA). Lymphocytes were initially gated by forward scatter/side scatter; secondary gates were set on the basis of staining with isotypic control monoclonal antibodies so that fewer than 1% of cells stained positive. Multiple control experiments were performed to validate the applicability of intracellular staining for cytokines, according to previously reported methods (29). Accordingly; we stained unstimulated splenocytes from naïve and immunized mice and lymphocytes that had been cultured for 6 h, with phorbol-12-myristate-13-acetate (PMA, ICN Biomedicals, CA, USA) at 50 ng/ml and calcium ionophore ionomycin (ICN Biomedicals, CA, USA) at 1 μM.

### Cytokine Measurements

Briefly, splenocytes obtained from naïve or EVs A-immunized mice were seeded at a concentration of 1.0 × 10^6^ cells per well in 96-well plates and incubated for 48 h at 37°C. IFN-γ levels were measured in culture supernatants by enzyme-linked immunosorbent assays (ELISA) using a commercial kit (eBioscience) following the manufacturer’s instructions. Samples were read at 492 nm (31).

### Statistical Analysis

The survival fractions were calculated using the product-limit Kaplan–Meier method, and differences between treatments were evaluated by log-rank statistic. For statistical comparisons, a one-way ANOVA with Tukey’s posttest was used. Differences were considered significant at *p* values <0.05 for all comparisons. The statistical analysis was performed using GraphPad Prism software 5.1 for Windows (GraphPad Software, San Diego, CA, USA).

## Results

### Isolation of EVs Derived from Ascites of Mice Bearing LBC Lymphoma

LBC lymphoma is an aggressive tumor. The terminal phase of the disease occurs between days 17 and 21 post-challenge (24). At 19 ± 2 days, when mice developed tumor ascites that were visually detectable, ascitic fluid was obtained, and the EVs were isolated. The average concentration of tumor cells in the ascitic fluid was 1.70 × 10^8^ ± 0.50 × 10^8^ cells/ml, and the average of EVs isolated, assessed as protein concentration, was 0.72 ± 0.06 μg/1.0 × 10^6^ LBC cells.

### Morphological Characterization and Protein Profile of EVs A

**Figure 1:**
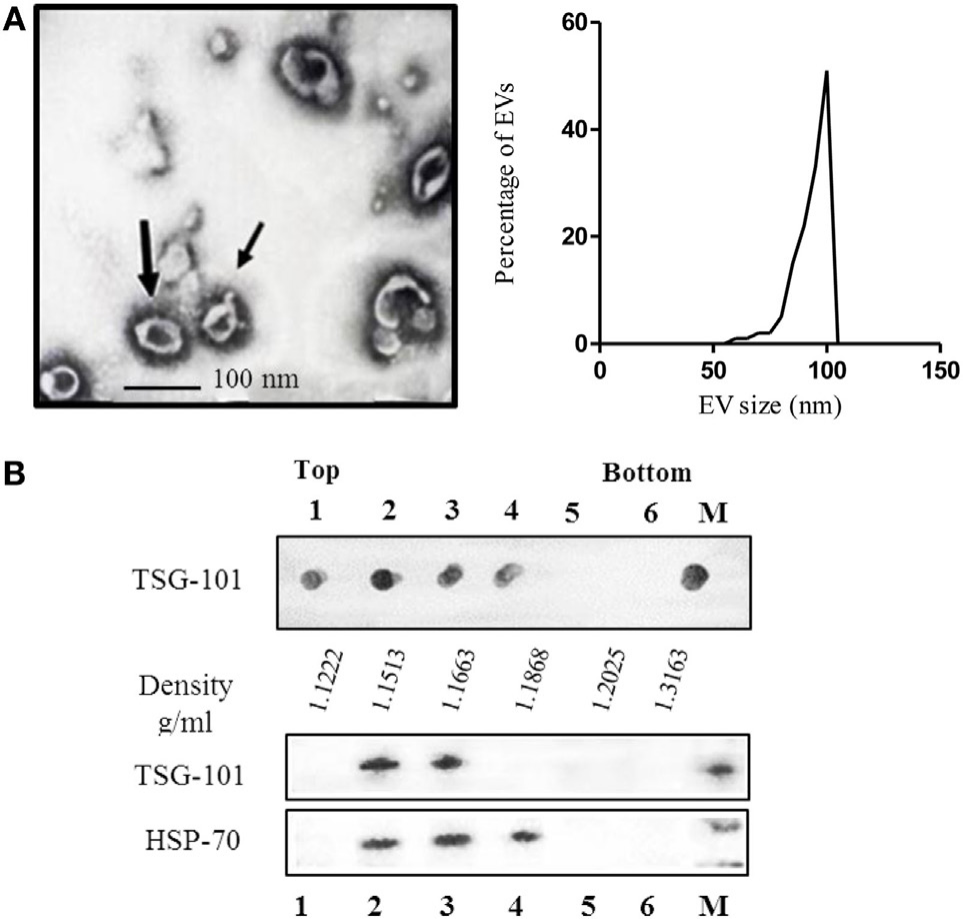
**(A)** Electron microscopy of purified extracellular vesicles (EVs) isolated from ascitic fluid samples, demonstrating the typical shape and size (60–110 nm) of exosomes, and graphic of the size-distribution of the vesicles measured with ImageJ software of at least eight micro-photos. **(B)** Characterization of EVs isolated by sucrose density gradient. The sample was loaded on top of a step sucrose gradient, and six fractions were collected and analyzed for exosomal proteins. Aliquots of fractions collected from the top of the gradient were analyzed by dot blot for TSG-101 and western blotting with antibodies against HSP-70 and TSG-101. M = pellet from 100,000 × *g* ultracentrifugation not submitted to a sucrose gradient sedimentation.

Ascitic fluid contains several types of EVs; therefore, we performed a characterization of the EVs obtained according to their morphological and physical properties as well as their protein profile. The morphological characteristics were analyzed by electron microscopy. As shown in Figure 1A, the EVs were not homogeneous, displaying the characteristic morphology and size (50–100 nm) of exosomal samples.

The density is another parameter widely used to characterize EVs (32, 33). It is well known that exosomes float on a continuous sucrose density gradient ranging from 1.15 to 1.19 g/ml. To characterize the nanovesicles obtained from the ascites, the vesicles were analyzed by their flotation in a continuous sucrose density gradient. After centrifugation, the density gradient fractions were analyzed by dot blot and western blot using a monoclonal antibody specific for the TSG-101 protein, which is a protein expressed by exosomes and associated with the endosomal origin of the nanovesicles. As seen in Figure 1B, the presence of EVs derived from ascites located at densities 1.1222, 1.1513, 1.1663, and 1.1868 g/ml was reveled by the expression of the TSG-101, analyzed by dot blot. Similar results were obtained by analyzing samples by western blot (Figure 1B). The lanes corresponding to fractions 2 and 3 (1.1513 and 1.1663 g/ml, respectively) revealed a band corresponding to TSG-101 (44 kDa) and lanes 2, 3, and 4 to HSP-70 (70 kDa), which is also a protein usually found in exosomes.

**Figure 2:**
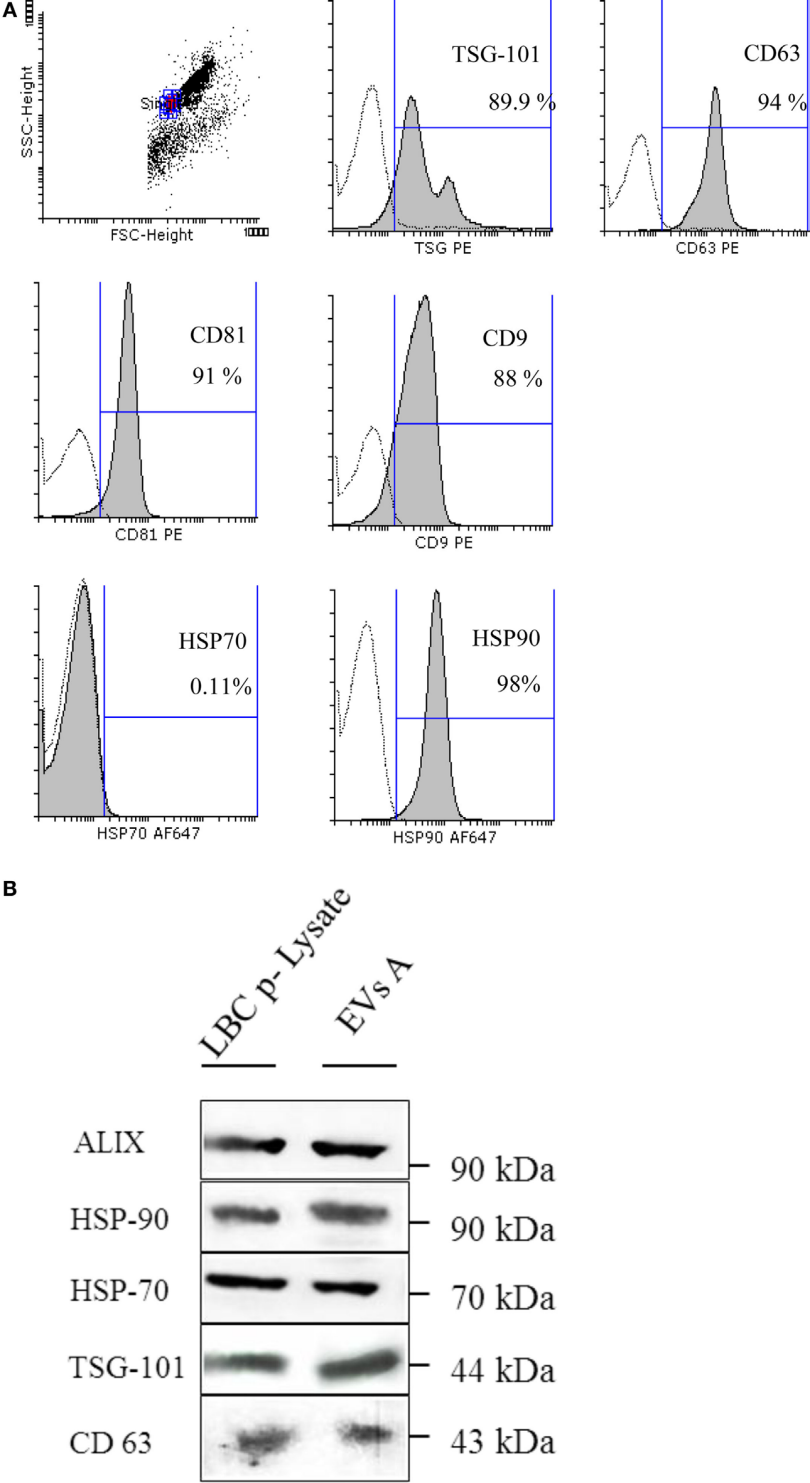
**(A)** Flow cytometry detection of surface molecules on extracellular vesicles (EVs). EVs were incubated with aldehyde–sulfate latex beads and stained with monoclonal antibodies or specific isotype-matched control antibodies. Analysis was performed on singlet gate of a forward scatter versus side scatter dot plot. Filled histograms represent exosome–bead complexes stained with specific monoclonal antibodies, the unfilled histogram represents isotype control antibodies. **(B)** Immunoblot protein analyses of EVs. EVs A (EVs isolated from ascites) and the LBC-cell lysate (LBC p-Lysate) were separated on a SDS-PAGE and electroblotted onto a nitrocellulose membrane. Samples were incubated with antibodies specific for heat shock proteins (HSP-70 and HSP-90) and specific markers for exosome as ALIX, TSG-101, and CD63.

To characterize the isolated nanovesicles, the protein profile of the tetraspanins classically used as exosomes markers, CD63, CD9, and CD81 and the endosome-associated proteins ALIX and TSG-101, were studied by flow cytometry and western blot (Figures 2A,B). By flow cytometry, it was demonstrated that 94% of EVs A express CD63 and almost 90% were TSG-101 positive; while 91 and 88% of the EVs A express the tetraspanins CD81 and CD9, respectively. These results were confirmed by western blot and in addition, the presence of ALIX and TSG-101 were demonstrated (Figure 2B).

Heat shock proteins are proteins commonly expressed in exosomes, and their localization may determine their immunological properties. Thus, one of the objectives was to analyze the localization of these proteins in EVs A. We first demonstrated that both HSP-90 and HSP-70 are expressed EVs A by western blot (Figure 2B). To further investigate the location of HSP on the outer surface of EVs A, we coupled EVs A to aldehyde–sulfate latex beads, and the expression of HSP-70 and HSP-90 was analyzed by flow cytometry. As shown in Figure 2A, HSP-90 was highly expressed on the surface of EVs A, while HSP-70 could not be revealed on EVs A by flow cytometry. HSP-70 expression could only be demonstrated by western blot (Figures 2A,B). Taken together, the results obtained by flow cytometry and western blot suggest that HSP-70 lies encapsulated in the exosomal *lumen*, and that HSP-90 is present on the surface. However, the presence of HSP-90 also in the EVs’ *lumen* cannot be ruled out.

**Figure 3:**
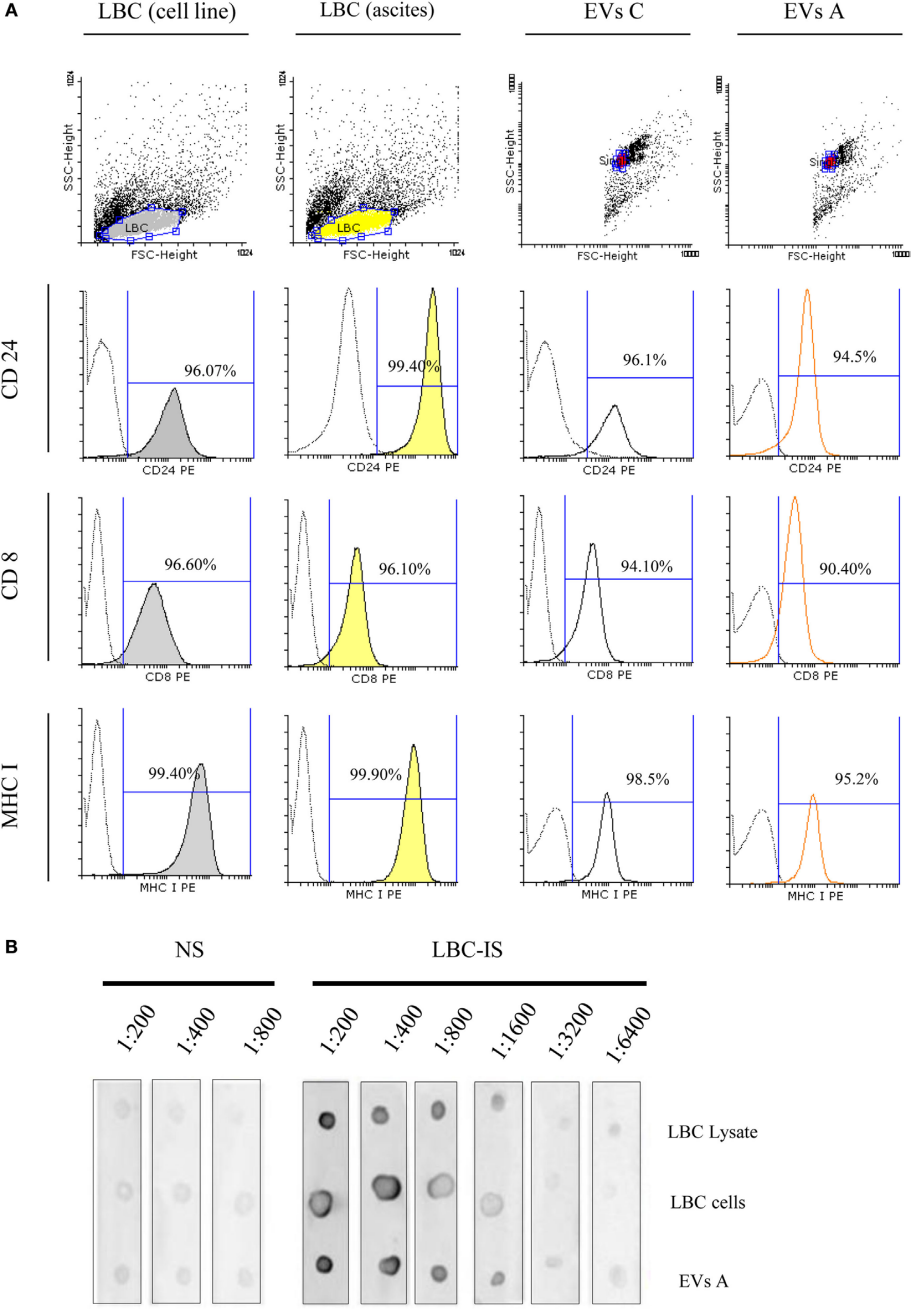
**Flow cytometry analysis of extracellular vesicles (EVs) and LBC cells**. **(A)** Samples were incubated with antibodies specific for CD24, CD8, and MHC I. Analysis was performed on singlet gate of a forward scatter versus side scatter dot plot. Filled histograms represent LBC cells stained with specific monoclonal antibodies, the unfilled histograms represent EVs–bead complexes, and dot line represents irrelevant-isotype-control antibodies. Graphs correspond to one representative of four independent experiments. EVs C, EVs isolated from LBC cell line. **(B)** Dot blot characterization of tumor-associated antigens in EVs A and LBC cells. Dot blot analysis of 1 μg LBC-cell lysates, 10,000 LBC cells (LBC), and 1 μg of LBC EVs. EVs proteins and LBC lysates proteins were separated on a SDS-PAGE and electroblotted to nitrocellulose. Blots were probed with sera from naïve (NS) or LBC lysate-immunized (LBC-IS) mice.

Due to their importance in the antitumor immune response, we next focused on those proteins with immunological relevance expressed on EVs A and compared them with the cells from which they derive (LBC ascites). Furthermore, we also included in the study LBC cells from *in vitro*-cultured-cell line and the EVs isolated from their conditioned medium (EVs C). By flow cytometry, we demonstrated that EVs A express proteins involved in antigen presentation such as MHC I, CD8, and the heat stable antigen CD24 (Figure 3A). As shown in the Figure 3A, LBC cells either growing *in vitro* or *in vivo* as well as EVs secreted by them, expressed similar levels of CD8, MHC I, and CD24. CD24 is a molecule expressed on thymocytes but not on mature thymic and peripheral T-cells and indicates the degree of malignancy in several tumors. CD24 is highly expressed on LBC as these cells are derived from an early T-cell precursor. The fact that more than 90% of EVs, either derived from ascites or from conditioned medium of LBC cells, express CD8 and CD24 suggests the tumor origin of the EVs A.

Based on our results, the small size of the EVs isolated and the expression of ALIX and TSG-101 together with the presence of CD63, CD9, and CD81 are strong evidence that the isolated nanovesicles are exosomes. Furthermore, we demonstrated that the amount of CD24 in EVs A correlated with the amount of CD8 and therefore most of the CD24-positive EVs are secreted by LBC cells.

### EVs A Express Tumor-Associated Antigens

Previous studies have shown a common protein pattern between exosomes derived from different cell types and proteins present exclusively in tumor-derived exosomes. We next investigated the existence of common antigens between LBC tumor cells and EVs A. LBC cells and EVs A were analyzed by dot blot, then probed with sera obtained from naïve mice (normal sera, NS) and mice immunized with LBC-cell lysate (immune sera, LBC-IS), as detailed in Section “Materials and Methods.” Upon incubating LBC cells and exosomes with IS, a high signal for LBC cells and exosomes was observed, obtaining titers of up to 1:6400 (Figure 3B). However, no signal was obtained when EVs A and LBC cells were incubated with NS. These results allow inferring that specific antibodies against LBC present in serum obtained from immunized mice do, in fact, recognize antigens on EVs A in a similar way. Therefore, it could be speculated that EVs A and tumor LBC cells share tumor antigens.

### EVs A Stimulate Specific Splenocytes *In Vitro*

Extracellular vesicles from tumor cells have proved either to suppress or to activate the immune response. Since EVs A were isolated from the ascites of mice in the terminal stage of the tumor development, where the immunosuppressive properties limiting the immune system’s ability to restrain the tumor may be present, we initiated the study by determining the effect of exosomes over the lymphocyte proliferative rate. Splenocytes obtained from mice immunized with LBC lysate as well as naïve mice were stained with CFSE, stimulated with CON A and cocultured with EVs A.

**Figure 4:**
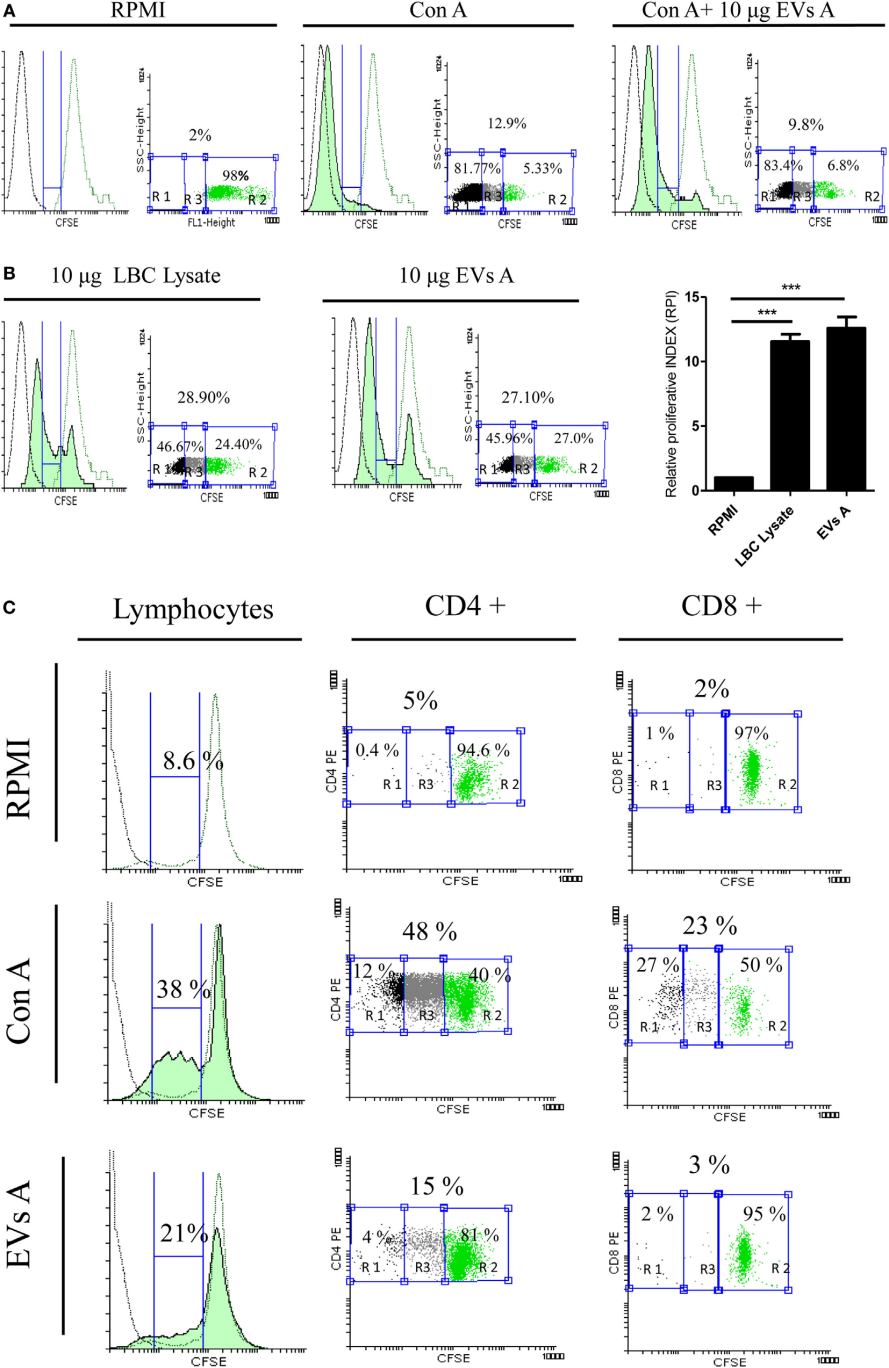
**Evaluation of the immune properties of EVs A *in vitro***. **(A)** Immunosuppressive capacity of EVs A. Splenocytes obtained from naïve mice were stained with carboxyfluorescein succinimidyl ester (CFSE) and incubated with 1 μg of concanavalin A (CON A) or 1 μg CON A + 10 μg EXO A in RPMI plus 5% fetal-bovine serum for 5 day. **(B)** Stimulatory capacity of EVs A. Splenocytes obtained from mice sensitized *in vivo* with LBC lysate were labeled with 5 μM CFSE and cultured for 5 days with 10 μg EVs A or 10 μg of LBC lysate (green-filled histogram). Flow cytometry histograms corresponding to non-stimulated splenocytes stained with CFSE used as negative control (green line histogram), unstained non-stimulated splenocytes (black line histogram). RPI, relative proliferative index, is defined as the ratio between the percentages of stimulated cells by the percentage of control cells. The percentage of proliferating cells was taken from G0 (not included) to the beginning of autofluorescence. The numbers in the graph correspond to the percentage of the gated region: R2, unstimulated CFSE stained cells; R1, autofluorescence; and R3, proliferating cells. Graphs correspond to one representative of two independent experiments. **(C)** Proliferation of CD4+ and CD8+ lymphocytes. Splenocytes obtained from mice sensitized *in vivo* with LBC lysate were labeled with 5 μM CFSE and cultured for 5 days with 10 μg EVs A or 0.5 μg CON A. Flow cytometry histograms corresponding to non-stimulated splenocytes stained with CFSE used as negative control (green line histogram), unstained non-stimulated splenocytes (black line histogram). The percentage of proliferating cells was taken from G0 (not included) to the beginning of autofluorescence. Graphs correspond to one representative of two independent experiments. Lymphocyte gate was determined by forward and side scatter. CFSE dye dilution was analyzed gating on CD4 and CD8 cell fractions.

The maximum proliferative rate was reached with CON-A-stimulated splenocytes regardless of whether or not they were incubated with EVs A. As shown in Figure 4A, when lymphocytes were stimulated with CON A and cultured with EVs A, 93% of lymphocytes displayed a left shift indicating a dilution of CFSE due to the proliferative response. Similar results were obtained when the cells were incubated only with CON A demonstrating that EVs A does not suppress the CON A-driven lymphocyte proliferation.

In a parallel experiment, the stimulatory capacity of EVs A was evaluated. Spleen cells obtained from mice previously immunized with LBC lysate and labeled with CFSE were cultured with EVs A or LBC lysate, used as a positive control. As shown in Figure 4B, after 5 days of culture, both EVs A and LBC lysate had similar, potent stimulatory capacities. The RPI was 11.54 ± 1.21 and 12.78 ± 1.39 for LBC-cell lysate and EVs A, respectively. A significant difference (*p* < 0.001) was observed for both treatments, when comparing each group with the unstimulated control (RPI = 1). In additional experiments, we studied the proliferation induced by the EVs A on CD4+ and CD8+ T-cells. As shown in Figure 4C, EVs A promote a specific proliferation of CD4+ lymphocytes (15%) and a slight proliferation of CD8+ (3%) when compared with non-stimulated splenocytes.

**Figure 5:**
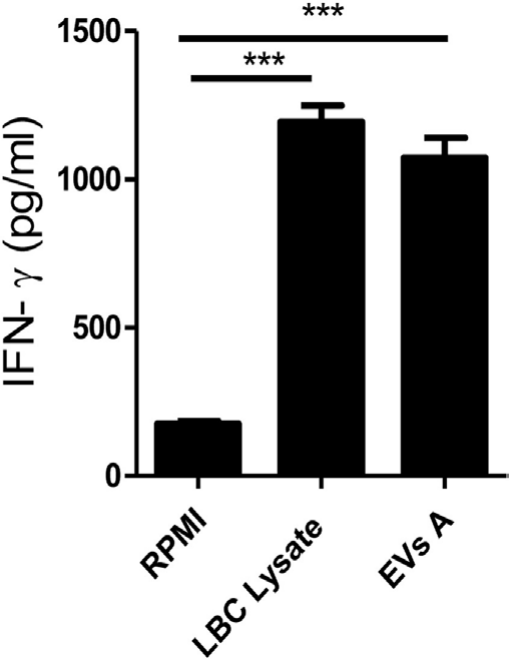
**IFN-γ production**. Spleen cells obtained from mice previously immunized with LBC lysate were incubated for 5 days at 37°C. Supernatants were then collected and IFN-γ levels were measured in culture supernatants by enzyme-linked immunosorbent assays. Error bars indicate SD of duplicate samples from a representative of two independent experiments. Asterisks indicate significant differences (***p < 0.001).

To further investigate the immune response induced by EVs A, the levels of IFN-γ were determined in culture supernatants of splenocytes obtained from LBC lysate-immunized mice, as an indication of a T helper 1 (Th1) response. EVs A induced the production of IFN-γ by splenocytes derived from immunized mice (Figure 5). Furthermore, the levels of IFN-γ were similar when splenocytes were stimulated with either EVs A or the LBC-cell lysate when compared with unstimulated splenocytes (*p* < 0.001).

### EVs A Vaccination Induce Protection against Tumor Challenge

To test the potential immunogenicity of EVs A *in vivo*, we analyzed the effects of a previous immunization with two doses of EVs A on the outcome of a subsequent challenge with live tumor cells. To this end, groups of BALB/c mice were prophylactically immunized on days −14 and −7 with 10 μg of EVs A, EVs C, with LBC lysate or with PBS, as positive or negative controls, respectively. To evaluate the immune response elicited, at day −1, serum samples from immunized mice were obtained and evaluated by dot blot for the presence of specific antibodies. On day 0, mice were challenged by an i.p. administration of LBC viable cells and the survival was recorded.

**Figure 6:**
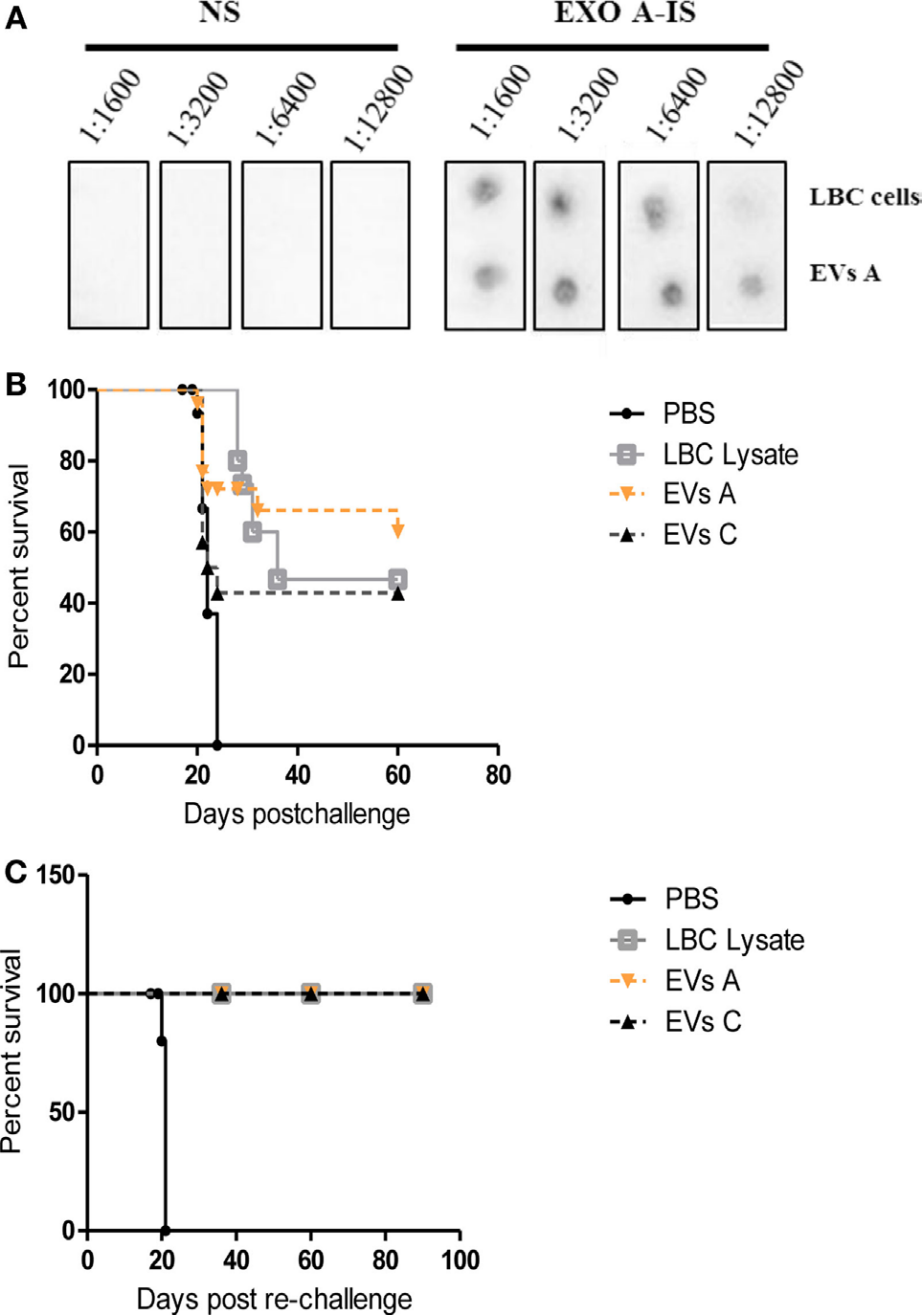
**Protective immune response against LBC tumor induced by EVs A immunization**. **(A)** Serum-specific antibodies against LBC cells. One microgram of exosomes or 10,000 LBC cells were spotted onto a nitrocellulose membrane. Membranes were blocked and incubated overnight at 4°C with different dilutions (from 1:1,600 to 1:12,800) of sera from naïve mice (NS) or from mice immunized with LBC lysate (LBC-IS). **(B)** Survival curves of mice immunized with EVs A. Immunocompetent naïve BALB/c mice were inoculated i.p. twice with 10 μg/mouse of EVs A with an interval of 7 days or with LBC lysate or EVs C. Seven days after the last immunization, mice were challenged with 1.0 × 10^6^ LBC tumor cells i.p. Phosphate-buffered saline (PBS) corresponds to the non-immunized control group, inoculated with PBS and challenged with the LBC tumor cells. *N* = 12/group. Graphs correspond to one representative of two independent experiments. **(C)** Survival curves of mice after tumor rechallenge. Mice vaccinated with EVs A or LBC lysate that had rejected the first tumor challenge were rechallenged with 1 × 10^6^ LBC tumor cells i.p. 30 days after the first tumor injection. *N* = 6/group. The curves corresponding to EVs A, EVs C, and LBC lysate overlap.

As shown in Figure 6A, the immunization with EVs A induced specific antibodies against both LBC tumor cells and EVs A. The antibody titer was slightly higher for EVs A than for LBC cells, while no specific antibodies were found in naïve mice sera (inoculated with PBS).

Approximately 60% of mice immunized with the EVs and challenged with LBC cells did not develop the tumor. Similar results were observed for mice vaccinated with LBC lysate; whereas all age-matched control mice developed tumors and died by day 22 post-challenge. Similar results were obtained immunizing mice with 10 μg of EVs isolated from LBC cells conditioned medium (EVs C) (Figure 6B).

To assess whether the immunization with EVs A could induce a long-term antitumor immunity against a second parent tumor challenge, mice that had survived over 30 days after tumor cell injection and naïve mice, used as control, were reinoculated with LBC cells i.p. All mice immunized with EVs A, EVs C, and the LBC lysate survived the lethal tumor rechallenge for more than 120 days, whereas 100% of mice in the control group died (Figure 6C).

To determine whether the immune response triggered by EVs A was tumor specific, mice surviving the first-LBC tumor challenge were rechallenged with the mammary adenocarcinoma LM3. As a positive control, naïve mice were inoculated with the LM3 tumor. All mice (those immunized with EVs A, EVs C, or LBC-cell lysate as well as the control animals) developed a palpable tumor on day 11 postinoculation. On day 40, when mice reached the final stage of tumor disease, all were sacrificed (data not shown).

### Immunization of BALB/c Mice with EVs A Generate IFNγ-Producing CD4+ and CD8+ Cells

To elucidate whether the immunization elicited a Th1 response, 35 days after tumor challenge we determined the levels of IFN-γ in splenocytes supernatant obtained from immunized mice and cocultured during 48 h either with EVs A or the LBC tumor lysate. This period was selected since the average death time for control animals (age-matched naïve) was 22 days.

**Figure 7:**
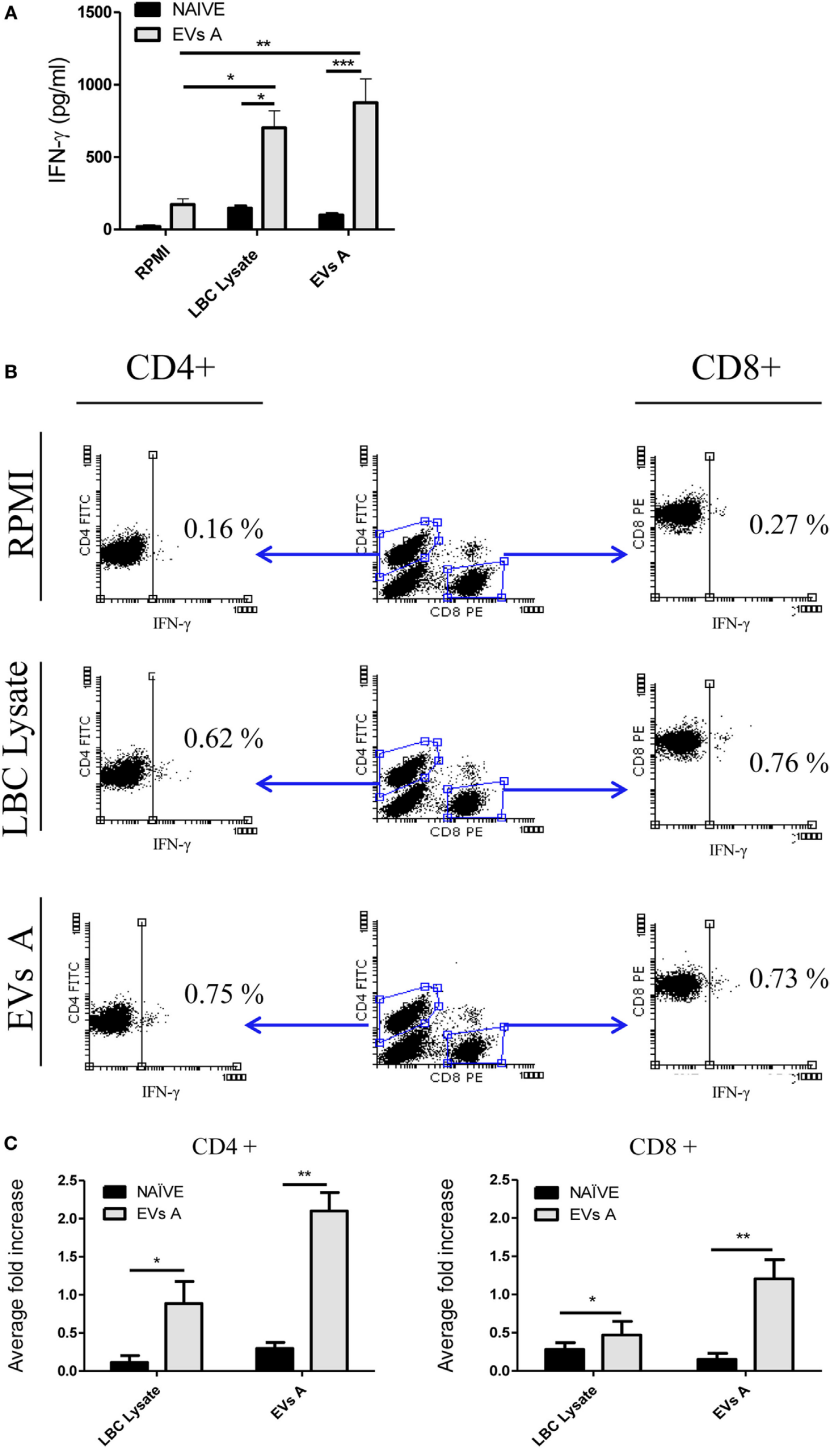
**Immune response induced in EVs A-immunized mice**. **(A)** IFN-γ levels in the supernatant of splenocytes from mice immunized with EVs. Spleen cells obtained from naïve mice or mice previously immunized with EVs A were incubated for 48 h at 37°C. Supernatants were then collected, and IFN-γ levels were measured in culture supernatants by enzyme-linked immunosorbent assays. Error bars indicate SD of duplicate replicates from a representative of two independent experiments. Asterisks indicate significant differences (*p < 0.05, **p < 0.01, and ***p < 0.001). **(B)** Intracellular staining for IFN-γ in lymphocytes from mice immunized with EVs A. Spleen cells obtained from naïve or mice previously immunized with EVs A were incubated for 48 h at 37°C. Double-color surface staining was first performed with conjugated mAbs fluorescein isothiocyanate -anti-CD4 and PE-anti-CD8, and then cells were permeabilized with saponin and stained with allophycocyanin-anti-IFN-γ. To analyze intracellular cytokine expression by CD4+ and CD8+ lymphocytes, a gate was first drawn around the lymphocytes in a dot plot of forward scatter versus SSC. A second gate was drawn around the CD4+ or CD8+ lymphocytes in the CD4 versus CD8 dot plot. Non-stimulated splenocytes were used as negative control (RPMI). Splenocytes cultured 6 h with PMA at 50 ng/ml and ionomycin at 1 μM were used and as positive control (data not shown). **(C)** Average fold increase in IFN-γ-producing cells. This index is defined as the ratio between % stimulated IFN-γ-producing cells and % IFN-γ-producing cells of unstimulated splenocytes. The % IFN-γ-producing cells used for the calculation was previously subtracted by the % of unstimulated control. Error bars indicate SD of duplicate samples from a representative of two independent experiments. Asterisks indicate significant differences (*p < 0.05, **p < 0.01, and ***p < 0.001). *N* = 6/group.

Figure 7A displays the levels of IFN-γ in the supernatant of splenocytes obtained from immunized mice cocultured during 48 h either with EVs A, the LBC-cell lysate, or medium alone. High levels of IFN-γ were detected in supernatants of splenocytes from immunized animals stimulated with either EVs A or the LBC lysate, as compared to unstimulated (EVs A *p* < 0.01 and LBC lysate *p* < 0.05) cells or those obtained from naïve mice (EVs A *p* < 0.001 and LBC lysate *p* < 0.05), suggesting the induction of a Th1 response.

To further characterize the cellular immune response induced, the intracellular IFN-γ staining was performed for specific T-cells subsets (Figure 7B). While naïve mice did not generate a LBC-specific T-cell response, mice immunized either with EVs A or the LBC lysate showed a significant increase of IFN-γ-producing CD4+ and CD8+ T-cells, when compared with those cells obtained from naïve mice (EVs A CD4+ *p* < 0.01; LBC lysate CD4+ *p* < 0.05 and EVs A CD8+ *p* < 0.01; LBC lysate CD8+ *p* < 0.05). An approximate twofold and onefold increase in IFN-γ-secreting CD4+ T-cells were induced when splenocytes from immunized mice were stimulated with EVs A and LBC lysate, respectively, compared with CD4−IFN-γ+ cells from naïve-control mice. Whereas an approximate 1- and 0.25-fold increase in IFN-γ-secreting CD8+ T-cells was found for EVs A and LBC lysate stimulated splenocytes, respectively, in immunized mice compared with CD8−IFN-γ+ from naïve-control mice (Figure 7C).

## Discussion

In this paper, we characterized EVs isolated from ascites of tumor-bearing mice in the late stage of the neoplastic development, and we gave an insight into the understanding of the relationship between tumor-associated antigens, immune relevant molecules, and their immune stimulating properties.

Ascites contain various types of cells and EVs; therefore, it was essential to confirm that the samples obtained were indeed exosomes (2). Our results demonstrated that LBC cells contained in ascites of tumor-bearing mice release EVs with biochemical and morphological characteristics that are those corresponding to exosomes. In fact, such EVs were found to express the exosomal-hallmarks CD9, CD81, CD63, and the endosomal sorting required for transport proteins, TSG-101 and ALIX. Besides, these EVs had a size and morphology and had the buoyant density in sucrose gradients reported for classical exosomes (2–4, 32).

Lambrecht et al. have demonstrated that exosomes purified from malignant effusions of patients with breast, ovarian, or lung cancer may be released not only by tumor cells but also by dendritic, B, and T-cells (34). The expression of CD24 and CD8 in 90% of EVs A excluded the presence of EVs from other cells such as dendritic, B, and T-cells present in malignant effusions of the peritoneum cavity (Supplementary Figure S1). Indeed, CD24 is expressed in a variety of malignancies including B-cell lymphoma, renal-cell carcinoma, nasopharyngeal carcinoma, hepatocellular carcinoma, bladder carcinoma, glioma, ovarian, and breast cancer. The expression of CD24 on LBC lymphoma correlates with the early stage of T-cell differentiation when the neoplastic transformation of the tumor cells took place (24). The high-level expression of CD24 has been correlated with the degree of tumor progression. Accordingly, CD24 is used as a cellular marker of progression for this lymphoma (24) as it is used for other tumors (35) associated with poor prognosis.

When analyzing the expression of HSP-70 and HSP-90, proteins also enclosed in EVs that are very well known for their adjuvant activity on immune responses, a different distribution of these proteins was observed when compared to LBC cells. While these proteins were expressed intracytoplasmatically in LBC cells (Supplementary Figure S2), a translocation to the surface of EVs A occurred for HSP-90. Indeed, this protein is normally found intra-lumen in exosomes (36, 37). Accordingly, we demonstrated an elevated expression of HSP-90 on the surface of EVs A, but not on the surface of EVs derived from the thymoma EL4 cells (Supplementary Figure S2). The surface expression of HSP-90 on LBC EVs may allow their binding to DCs-surface receptors such as CD91, inducing cell maturation. This atypical cell-surface expression of HSP-90 may also contribute to a tumor-specific cellular immunity, as demonstrated for mast cell-derived exosomes (38). Even more remarkable, Spisek et al. have demonstrated that the induction of HSP-90 on the surface of human myeloma cells enhances the uptake of tumor antigens by DCs, leading to the induction of antitumor immunity (39).

Additionally, EVs A express immunologically relevant molecules on the surface such as CD8 MHC class I and LBC tumor-specific antigens. These antigens present on EVs A are recognized *in vitro* by memory lymphocytes generated *in vivo* with LBC lysate. In fact, we have demonstrated by means of lymphocyte proliferation assay that LBC exosomes are stimulators of the immune system, inducing a potent Th1 response with tumor-specific CD4 and CD8 expansion and the production of IFN-γ. Even more, LBC EVs exert an *in vivo* immune stimulation that is both cellular and humoral. The immunization with LBC EVs induced an immune response featured by specific IFN-γ CD4+ and CD8+ secreting T-cells and the synthesis of antibodies against LBC cells.

Contrary to the findings for other exosomes present in malignant effusions with suppressors properties (40, 41), EVs released by this T-cell lymphoma induced a potent immune response and could be use as acellular antigens. In fact, we demonstrated that EVs A induced a significantly high immune response since it allowed mice post-challenge survival. Moreover, the immunization with EVs A conferred a long-lasting memory response, since it induced protection against a second challenge with the LBC tumor.

The advantage of employing EVs and exosomes for cancer immunotherapy lies in the use of acellular tumor-associated antigens that are indistinguishable from those expressed on the cell from which they derive. Besides, this stimulation would increase efficiency, since tumor antigens are concentrated in EVs. Moreover, they are highly reproducible, easy to store, safer, and more stable than irradiated cells.

Our results revalue the use of nanovesicles as acellular antigens to be used in immunotherapy against cancer. It is of great importance to define the molecular composition of the EVs and exosomes together with its immunomodulatory properties for each tumor, prior to its use as therapy, because nanovesicles have the ability to activate or inhibit the immune system. EVs therapy offers a promising alternative to a better immune strategy for the development of cancer vaccines.

## Ethics Statement

The protocol was approved by the Institutional Animal Care and Use Committee (CICUAL) guidelines of University of Buenos Aires Medical School.

## Author Contributions

Conception and design: FM, LH, JT, and CM; execution of experiments: FM, LH, JT, FC, MPDS, and RT; acquisition of data: FM. FC, MG, and RT; analysis and interpretation of data: FM, CM, AV, MG, CW, and CM; drafting of the manuscript: FM and CM; critical revision of the manuscript: MG, CW, and CM; obtained funding: CM and CW; study supervision: CM; final approval of the version to be published: CM, MG, and CW; agreement to be accountable for all aspects of the work: CM.

## Conflict of Interest Statement

The authors declare that the research was conducted in the absence of any commercial or financial relationships that could be construed as a potential conflict of interest. The reviewer PH and handling editor declared their shared affiliation, and the handling editor states that the process nevertheless met the standards of a fair and objective review.

## Supplementary Material

Figure S1 **Expression of CD3, CD4, CD8, B220, and CD24 on cells obtained from the ascites of LBC tumor-bearing mice**.

Figure S2 **Expression of HSP-90 in LBC and EL4 tumor cells and EVs secreted from these cells**.
