# Supplementary figures and images for: Exosomes Isolated from Ascites of T-Cell Lymphoma-Bearing Mice Expressing Surface CD24 and HSP-90 Induce a Tumor-Specific Immune Response
Source: Front Immunol. 2017 Mar 16;8:286. doi: 10.3389/fimmu.2017.00286 (PMC5352668; doi:10.3389/fimmu.2017.00286)

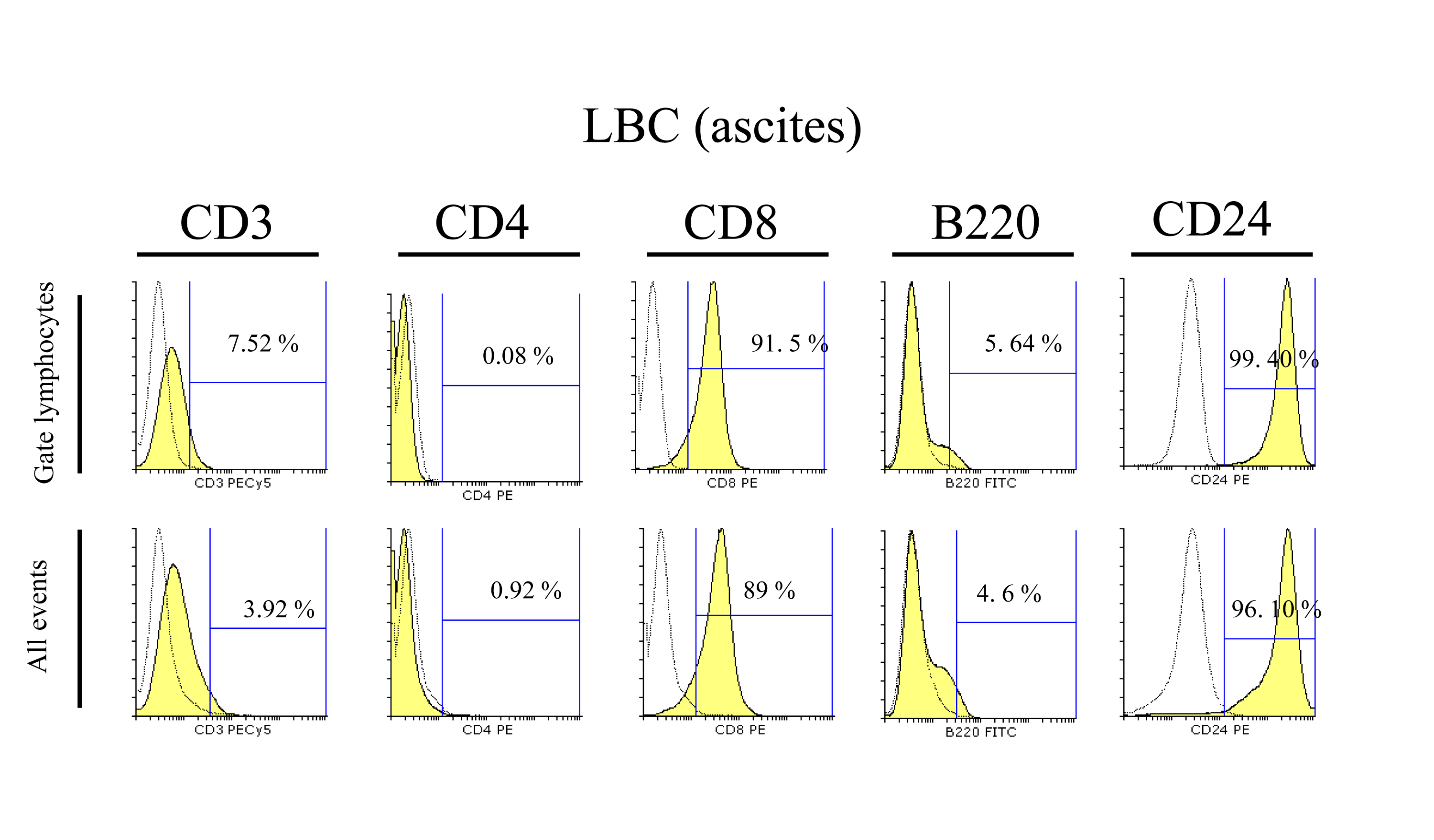

Supplement: Figure S1 — Expression of CD3, CD4, CD8, B220, and CD24 on cells obtained from the ascites of LBC tumor-bearing mice. [file Image_1.tif]

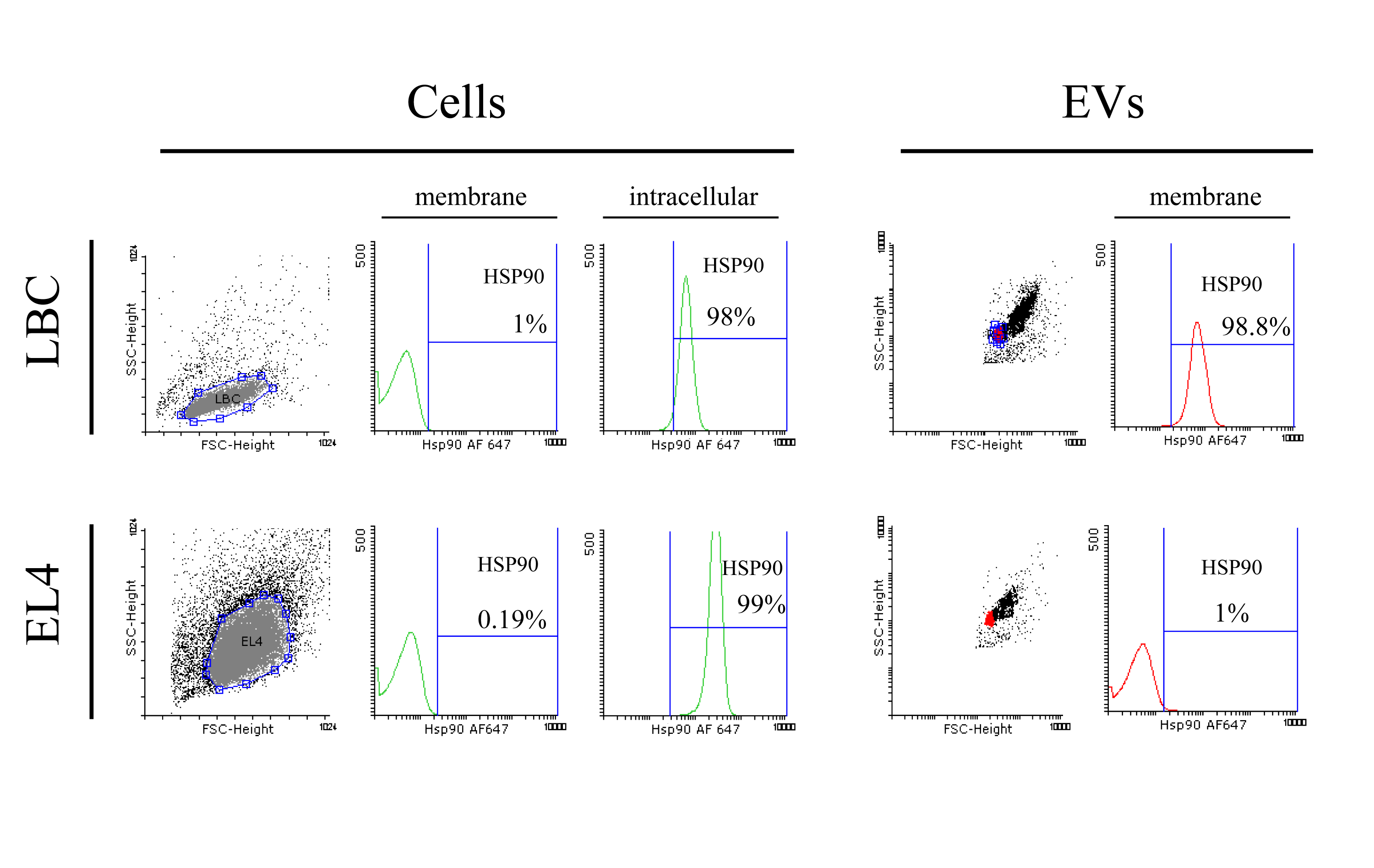

Supplement: Figure S2 — Expression of HSP-90 in LBC and EL4 tumor cells and EVs secreted from these cells. [file Image_2.tif]
